# Local Choices: Rationality and the Contextuality of Decision-Making

**DOI:** 10.3390/brainsci8010008

**Published:** 2018-01-02

**Authors:** Ivo Vlaev

**Affiliations:** Warwick Business School, University of Warwick, Coventry CV4 7AL, UK; Ivo.Vlaev@wbs.ac.uk

**Keywords:** rationality, judgment, decision-making, inference, preferences, context effects

## Abstract

Rational explanation is ubiquitous in psychology and social sciences, ranging from rational analysis, expectancy-value theories, ideal observer models, mental logic to probabilistic frameworks, rational choice theory, and informal “folk psychological” explanation. However, rational explanation appears to be challenged by apparently systematic irrationality observed in psychological experiments, especially in the field of judgement and decision-making (JDM). Here, it is proposed that the experimental results require not that rational explanation should be rejected, but that rational explanation is *local*, i.e., within a context. Thus, rational models need to be supplemented with a theory of contextual shifts. We review evidence in JDM that patterns of choices are often consistent *within* contexts, but unstable *between* contexts. We also demonstrate that for a limited, though reasonably broad, class of decision-making domains, recent theoretical models can be viewed as providing theories of contextual shifts. It is argued that one particular significant source of global inconsistency arises from a cognitive inability to represent absolute magnitudes, whether for perceptual variables, utilities, payoffs, or probabilities. This overall argument provides a fresh perspective on the scope and limits of human rationality.

## 1. Introduction

A paradox appears to lie at the hearts of psychological explanation. The explanation of thought and behaviour in rational terms is central to psychological theorising across a vast range of psychological domains and theories, ranging from the rational analysis of memory, categorisation or reasoning [1,2] expectancy-value theories of attitude formation [3], ideal observer models in perception [4] mental logic [5] to probabilistic frameworks [6] among many others. More broadly still, rational choice theory is a paradigm of social scientific explanation, ranging across not just psychology, but also economics, sociology and political science. In addition, our intuitive, folk psychological, explanation of each other’s behaviours in terms of beliefs, desires, and other propositional attitudes appears essentially to presuppose the assumption of rationality, which links our beliefs together, and forges connections between our beliefs, desires and actions [7,8,9]. However, when human rationality is exposed to the glare of the psychological laboratory, it appears to be astonishingly frail: people appear to be subject to elementary and systematic errors in their reasoning, judgement, and decision-making. The rationality assumptions that appear to be central to so much psychological explanation appear to stand up poorly to rigorous experimental scrutiny.

This perplexing state of affairs has been a principal motivation behind empirical research on rationality for more than half a century, and a variety of theoretical directions has been proposed. One approach to reconciling “rational theory” with “irrational data” is to suggest that perhaps the data is not, on inspection quite as irrational as it may seem (e.g., [10,11,12])—perhaps an inappropriate rational theory has been applied, or perhaps apparent irrationality in the data arises not from any irrationality of thought, but from more superficial problems people may have in the potentially noisy process of translating thoughts into, for example, responses on scales, and concerning quantities (e.g., probabilities) that may be poorly understood (e.g., [13,14]); and perhaps laboratory tests of rationality can be downplayed because they lacked “ecological validity” [15].

A second perspective is to argue that it is our theories of rationality that need to be challenged and perhaps even abandoned [16]. Theories of rationality typically focus on the internal coherence of beliefs, values and actions, but perhaps such coherence is of marginal significance for cognitive function—perhaps, for example, we should focus instead on correspondence with reality, rather than coherence between beliefs, or the ability of a person to deploy decision-making strategies that are effective in their ecological environment, rather than justified in terms of underlying beliefs and desires (e.g., [17,18]). 

This paper focuses on a “third way”: namely that rational explanation is of central importance to understanding the mind and cannot be discarded, but that such explanation is local to a specific context. From this point of view, rational explanation in psychology captures the inferential relations between the beliefs, values, and actions that are created within a context; and laboratory reasoning and decision-making “anomalies” show that beliefs, values, and actions are not stable between contexts. 

We will argue that relevant beliefs, values and actions are created on-the-fly in a given context (this viewpoint is closely related to the constructive view of preferences and probability (e.g., [19,20]). When presented with a context, a person has to construct relevant preferences and beliefs, and will create and evaluate arguments in order to do so. For example, when confronted with an array of, say, cameras, a potential consumer cannot simply consult a pre-existing inventory of preferences for different camera types and features, or a pre-existing set of beliefs about how likely each feature is to be useful in practice, how likely each camera will break down or become obsolete, and so on. Instead, the process of consideration requires creating arguments, based on background knowledge, recommendations, prior experience, and so on, that partially address these questions, and raise further relevant questions. Such arguments are, of course, by their very nature, to be explained in terms of rational explanation. However, different contexts will highlight different arguments and different further questions, and will lead different beliefs, values and actions to be constructed. Of course, sometimes, we are able to find arguments that create partial links between contexts (e.g., last time I bought a TV, I bought Brand X, and it worked out well, so perhaps I’ll buy a Brand X camera), but such inferential links will be sparse and often difficult to create.

From this perspective, then, preferences, beliefs, and actions are created piecemeal, as the context demands. Rational explanation as central to the process: it underpins the arguments that allow us to make such constructions. However, different contexts will prompt the construction of different preferences, beliefs or actions.

Our argument proceeds as follows. In the next section, Rational Explanation, we review the wide variety of rational approaches to understanding psychological phenomena. We then argue, in Rationality is Local, that such explanation applies locally within contexts, rather than globally across a “context of all contexts” (if such a notion even make sense). We then illustrate this viewpoint, focusing on the study of choice. We introduce the issues in Local Rationality in Choice I: Background. Next, in Local Rationality in Choice II: Empirical Data, we stress that the empirical data indicate local rationality within a context, but not between contexts. Local Rationality in Choice III: Psychological Theories provides taxonomy of psychological models of choice, which indicates that there is a wide variety of ways in which the local nature of rationality can be captured, and, by capturing the aspects of the context, these models also provide accounts of how choices change when the context shifts. Finally, we draw out broader implications of this viewpoint in the Discussion.

## 2. Rational Explanation

Rational choice theory, the dominant theoretical paradigm in many areas in the social sciences, is a framework for understanding and often formally modelling social and economic behaviour. The theory is often conveniently formulated to assume that actors have perfect information, a perfect grasp of their objectives, and the perfect ability to use that information to make decisions to further their objectives (see [21]). Many sub-disciplines within psychology use rationality assumptions of various kinds. Rational analysis paradigm in cognitive science [1,11,22], including theories of motor control [23,24] and neural information processing [25], postulates that agent’s goals and environmental constraints determine the optimal behaviour in a particular context; rational principles still play a key role in determining what behaviour is optimal (given the agent’s goals and the environmental structure). For example, statistical decision theory successfully predicts optimal action selection in sensorimotor control [23], in which estimated noisy signals in our sensory and motor systems are combined with knowledge of the potential costs or rewards of different action outcomes.

In the psychology of reasoning, the cognitive system is often seen as a logical inference machine. The paradigm example of such models is “mental logic” theories in the psychology of reasoning, which regard the syntactic proof theory for logic as the basis of the algorithms that implement logical inference in the mind (e.g., [5,26,27]). Alternatively, Bayesian models of reasoning and inference (e.g., [11,20,22]) view the mind as a probabilistic inference machine—although perhaps one that performs only approximate calculations (see [28]).

In behavioural ecology, concepts from evolutionary biology predict how an animal should respond to risk if it is maximising its fitness. In this case, the normative approach (e.g., risk-sensitivity theory) is unique in predicting a shift in a subject’s response to risk as a function of its resource budget [29]. In animal cognition and behaviour, optimal foraging theory predicts foraging decisions in birds and mammals (see [30] for a review).

In social psychology, a highly influential model of attitudes and behaviour, Fishbein and Ajzen’s [3] *theory of reasoned action*, which was later superseded by the *theory of planned behavior* [31], the *theory of interpersonal behaviour* [32], and the *social-cognitive theory* [33], are all examples of *expectancy-value models* of attitudes, which are related to *subjective expected utility* in economics [34]. In models of this type, attitudes (and corresponding behaviours) are functions of beliefs (i.e., the subjective probability) for which the behaviour or object of interest will produce a given outcome and the subjective (positive and negative) values of the outcome.

A similar approach is taken in health psychology. According to the prominent *health belief model* [35], people evaluate health-related actions and states on the basis of subjective assessment of susceptibility (risk of getting the health condition), severity (seriousness of the health condition and its possible consequences), barriers (factors that facilitate or prevent adoption of the promoted behaviour), and benefits (positive consequences of adopting the behaviour). Similarly, the more recent *protection motivation theory* [36] proposes that two processes determine protective behavior—threat appraisal (beliefs about one’s vulnerability to a disease and about the severity of a disease) and coping appraisal (beliefs about the efficacy and costs of a recommended response).

Even the increasingly popular *dual-process models* of reasoning and judgment in cognitive and social psychology still preserve rationality assumptions when behavior is understood as a function of two distinct processes or systems (or sets of systems): evolutionarily older ‘System 1’ processes described as automatic, uncontrolled, effortless, associative, fast, unconscious and affective, and more recently, characteristically human ‘System 2’ processes described as reflective, controlled, effortful, rule-based, slow, conscious and rational (see [37,38,39]), for surveys of the research on dual-process theories in psychology). The two systems also differ in the depth of cognitive processing of information—one system is more ‘superficial’ and heuristic, while the other system provides more systematic and ‘deeper’ analysis (dual-process theories can be traced back to William James who believed that there were two different kinds of thinking: ‘associative’ reasoning and ‘true’ reasoning [40]. Dual process theories can be found in social, personality, cognitive, and clinical psychology (e.g., in cognitive psychology, attention and working memory have also been conceptualised as relying on two distinct processes [41]). 

Rational choice is the predominant paradigm in other social sciences such as economics, law, political science, criminology, sociology, and philosophy [21,42,43,44,45,46,47,48]. In economics, for example, Gary Becker’s [49] research on human social interactions has had many implications for the family, such as for the marriage market, divorce, fertility, and social security. Becker argued that such decisions are made in a marginal-cost and marginal-benefit framework (e.g., he concluded that wealthier couples have higher cost to divorce and thus a lower divorce rate). Similarly, theories of risky decision-making are based on *expected utility theory* [34], which plays a key role in theories of rational choice (for a review see [50]). The assumption has then been that, to an approximation, people do make decisions as they ought to, that is, this version of rational choice theory can be viewed as a descriptive, as well as a normative, theory of human behaviour [51,52]. In particular, given some relatively weak axioms (such as transitivity) concerning a person’s preferences over arbitrary risky prospects (i.e., gambles, where a number of outcomes is each associated with a probability), it is possible to derive a utility function maximized or optimized by that person. This utility function is not assumed to be explicitly represented by the decision maker—nonetheless, a person’s preferences between different gambles (or riskless options) can be viewed as “revealing” their implicit utility function—and, if the consistency assumptions hold, these utilities can predict preferences across other gambles or consumption bundles.

Rational explanation may also be made more complex by allowing that agents may have imperfect cognitive abilities with which to process the available information. This dimension of “bounded rationality” [53,54] is the focus of a large literature in psychology, economics, and other social sciences. At a broad level, bounded rationality is a mathematical necessity: the sophisticated calculations involving probability and decision theory that are invoked in economic analysis are known to be, in general, computationally intractable, and therefore, presumably, beyond the computational powers of the brain [2,55,56,57].

As a result of such limitations, the cognitive system is constrained to work with limited information. For example, even though decision-making can be viewed as being analogous to visual perception, which is often viewed as optimal—being approximated by *Bayesian inference* (see [4,58,59,60]), visual illusions such as the *Necker cube* [61,62] or the *duck-rabbit illusion* ([63], p. 312; see also [64]) suggest that the same input might trigger different perceptions due to selective processing of sensory information. Similarly, in a decision problem, humans are limited to the amount of information that they can absorb [65,66,67]. For example, in a multi-attribute choice problem, we might be able to pay attention to one dimension at each moment [68], which reduces the process into a sequence of binary, dimension-wise comparisons, and thus we cannot establish stable trade-offs [69]. Indeed, different processing operations in the brain (e.g., for perception, motor control, memory, reasoning, and language) each have very limited capacity and are almost serial [66], and also perform statistical inferences of some kind (e.g., see [4,6,70,71]).

## 3. Rationality Is Local

Our key proposition is that, as a result of various cognitive, biological, and environmental limitations, *local rationality* is the human tendency to make local, contextualised inferences, which could lead to global inconsistency in behaviours across contexts (situations, domains). This seems to reflect a basic property of human cognition that applies right across psychology, from the basic psychophysics of sound perception right through to high-level cognitive processes in judgement and decision-making. Here, we explore the basic underpinnings of this cognitive limitation and also reveal the implications for psychology, economics and public policy, where we suggest they raise important questions for the central methodologies used to measure and derive human preferences.

In this article, we argue that rationality assumptions typically hold *locally*—i.e., within a context, but not globally across contexts. It is as if we have a big library in our heads and we can only look at a few books at a time when we make up our mind on a specific issue. This view also preserves the picture of humans as creatures striving to make sense of, and hold consistent beliefs about, the world and their behaviour in it [72], which is exemplified in our need to hold consistent or non-contradictory beliefs. For example, when our behaviour and our self-beliefs are in conflict, it is often our beliefs and attitudes that get adjusted—so they are consistent with our behaviour [73,74]. 

The cognitive system may attempt to maintain local consistency, by following rational principles. If the information available to the ‘processor’ is limited by processing resources, time constraints, motivation, and memory accessibility (see [53,54,75,76]), then this process will still produce biased inferences—determined by what information is immediately available from the senses or from memory. For example, many *attribution biases* [77] share the common tendency to over-value dispositional (i.e., personality-based) explanations for the observed behaviours of others, while under-valuing situational explanations for those behaviours (e.g., self-serving bias occurs when people attribute their successes to internal/personal factors but attribute their failures to situational factors beyond their control, [78]. This interpretation makes sense locally unless we integrate evidence from our experiences on other similar situations, look for unseen causes and less-salient factors, asking oneself how one and/or most people would behave when put in the same situation, taking into account the fact that we may unconsciously want to avoid regret and disappointment, and so on, which is all very costly, takes time, and even then it may not be properly integrated into the inference process because of attentional limitations and constraints on the amount of information we can hold in our working memory [79]. Note that what is known as the *bounded rationality* paradigm [53,54] also proclaims that the sophisticated calculations invoked in decision theory and economic analysis are known to be beyond the computational powers of the brain. However, even though global information is hard to obtain and integrate, if people are able, or enabled, to do so, their inferences might be accurate or approximately rational (see [80]) for a given set of circumstances (although still very unlikely that their rationality can be truly global after all). 

Similar processes occur in other domains, such as affective forecasting, where people tend to over-predict the duration of affective reactions to future events, which is attributed to *focalism*—people focus too much on the event in question and not enough on the consequences of other future events, but asking people to think about other future activities reduces the bias [81]. Buehler, Griffin, and Ross [82] found that people underestimate how long it will take them to complete future tasks—the so called *planning fallacy*, which is explained by focusing too much on the future task and not enough on past experiences with similar tasks (see also [83], for how drawing attention to unseen and unforeseen pieces of information, causes or consequences, can help people make less biased judgments in other domains such as probabilistic forecasting).

In summary, the evidence suggests that whatever information is currently or locally available determines our beliefs, values and actions. This process can initially rely only on sampling local external information. For example, Bem’s [84] *self-perception theory* proposes that we form our attitudes, opinions, and other internal states by observing our behaviour and concluding what attitudes must have caused them. The theory suggests that people induce attitudes without accessing internal cognitions and moods, which is done by reasoning about one’s own overt behaviours rationally in the same way a person attempts to explain others’ behaviours [85]. This process is illustrated in a classic experiment, in which students were invited to do a rather boring task and then paid either $1 or $20 to persuade others to do the task [86], and those participants that paid more found it more aversive and were less likely to do it again because they used the amount paid as a justification for their attitudes towards the task (e.g., “if I was only paid $1, it can’t have been too bad”), not their actual experience. Even though this result is interpreted as an example of cognitive dissonance reduction (between the boring experience and the low $1 pay signalling the task is not bad), the study also demonstrates how people are relying on local rationality—it is the local cues that modify beliefs (e.g., being paid the $20 dollars not $1). Thus, instead of endorsing ‘global’ beliefs and attitudes about what is universally good or just for them, humans tend to engage in such local, situational inferences, which are, we claim, the key mediating mechanisms contributing to such tendencies (of course, after many repetitions in various domains and contexts, such attitudes might become universally endorsed, partially due to enhanced memory traces, but this can hardly apply to most everyday occasions where attitudes are expressed in decisions and behaviours).

We argue that rationality is local: it concerns relationships between particular beliefs, values, and actions in a local context. Of course, we’re not entirely in the grip of the mandatory context in which we are placed. Indeed, one function of deliberative thought appears to be to help us draw on how we have thought and acted in previous contexts. Thus, for example, while in Festinger and Carlsmith’s study, people who were paid a large amount of money to signal enjoyment of the task, a presumed future participant, and who hence infer that it must have been a very negative experience, also bring influence, to some degree, by salient experiences of similarly repetitive tasks (e.g., and being influenced by, for example, whether they have, in the past, found repetitive task pleasant or aversive). Such past experiences will have arisen in perhaps very different local contexts, and actively comparing present and past experience itself provides yet a further local context. In addition, in this context, a person may attempt to resolve the unexpectedly high payment with the fact that they have previously enjoyed repetitive tasks, perhaps by concluding, say, that it is the purposeless nature of the ‘peg-turning’ task, rather than its repetitive character, which is particularly unpleasant). However, the fact that local context can be linked together, to create a fresh context of deliberation, does not diminish the fact that reasoning and rationality are local.

It is tempting to suggest that a fully rational individual should link up *all* their beliefs, values and actions across *all* contexts—and that, by doing so, a person would be not merely locally, but globally rational, where global rationality is a ‘limiting case’, where all contexts are linked together. However, this ‘limiting case’ view of global rationality may not even make sense. Imagine that we could record both a person’s utterances and their unspoken thoughts (even assuming these could be given a propositional content). At one moment, a person says “It is raining” and at another “It is not raining”. This appears to be the starkest possible example of global inconsistency. However, it may, of course, just reflect a change in context: the person looks out of the window and disconfirms their initial conjecture; or the weather changes; or, more subtly, *meaning* has shifted (at one moment, the person was thinking of rain at the live soccer game, at the next, their own neighbourhood), or the contrast might have changed (at one moment, the person was wondering ‘Is there rain or no form of precipitation?’ and at the next ‘Is this rain or sleet?’ Even to *assess* global consistency across contexts requires being able to assign a ‘canonical’ and context-free meaning of each thought (specifying time, place, the referent of all terms, and so on, rather akin to the philosopher Quine’s, [87], notion of “eternal” sentences, true once and for all); and also to factor any relevant changes in the evidence on which belief is based. It is by no means clear that this attempt to translate words (and perhaps thoughts) into a completely context-independent language in which global consistency could be evaluated—indeed, there are reasons for skepticism that this is possible, although they are beyond the scope of this paper (e.g., [88]). If this is right, local rationality, i.e., within a context, is the only theoretical option available. 

We have so far argued for the local nature of rationality in general terms. We now focus on a particularly important illustrative example: the local rationality of choice.

## 4. Local Rationality of Choice I: Background

The local rationality hypothesis makes the intriguing, and counterintuitive, prediction that people have no internal value or utility scales, and instead are only able to make relative comparisons between items, and, moreover, that even these comparisons may not be invariant by may, instead, depend on local context. Thus, in a particular context (e.g., faced with a specific array of options), one may prefer A to B, but no meaningful “value” or “utility” can be assigned to either option. According to many theoretical accounts, instabilities of values may be attributed either to imperfect information (agents do not know what the consequences of A and B will be), or bounded rationality (even if the information is available, calculations concerning the probabilities of those consequence, and their utility, may be too complex to be carried out). However, we consider the more radical position—that the problem with conventional rational choice explanation is not that values and attitudes are well-defined but difficult to determine, but that they are not well-defined at all. Nonetheless, we shall see that this perspective is actually surprisingly consistent with large parts of standard rational choice theory.

The framework described here derives some universal implications for most descriptive models of choice, across domains and disciplines in behavioural sciences, which boils down to the core principle that decisions are relative, in a specific way, to what is embedded in the local context. A plausible account is summarised by the claim that because people lack computational and time resources to integrate all relevant information from memory or the world, they have poor notions of absolute cooperativeness, risk, or utility, and, instead, humans can only make judgments and decisions in relative terms—analogously to the presented psychophysical and cognitive theories of perception and judgment of information about magnitudes representing intensities of stimulus attributes (see [89] for a review of the evidence).

The approaches discussed so far are descriptive and not prescriptive, i.e., apply at the algorithmic or implementation level in Marr’s [61] terminology, though the resulting behaviour may be eventually adaptive. Therefore, we also need to discuss models that apply to the computational level in Marr’s terminology, which maximise an objective function (e.g., reward, value, etc.). There are two recent models that provide a more explicit justification of why context sensitivity is ‘rational’. The first is based on a ‘divisive normalization’ algorithm that computes a ratio between a given neuron’s response and the summed activity of a larger pool of neurons [90,91] and proposes that deviations from optimality arise from biological decision mechanisms that have evolved to maximize choice performance within innate biophysical constraints. Basically, sensory processing as well as decision-related brain areas utilise specific computations such as divisive normalization (division by the pooled activity of other neurons which drives spatial contextual modulation) to maximize information coding in constrained neural circuits. These adaptive computations implement a relative value code that the authors use to explain the characteristic context-dependent nature of behavioural violations of classical normative theory. 

The second perspective [92,93] is based on Bayesian inference, and more specifically on the notion that choices minimize surprise (or what is known as ‘prediction error’). This framework assumes that agents perform accurate inference about reward, and that context sensitivity of choice emerges from this process. The authors describe a unifying theory, referred to as a ‘Bayesian model of context sensitive value’, which explains between-context and within-context effects, in single- and multi-attribute decisions. This approach assumes that the brain builds a generative model of reward within a context and employs Bayesian inference to invert the model and compute the average reward for attribute on the basis of observing different reward amounts of options that are available in a given context (i.e., the inference forms a posterior belief about the underlying reward distribution every time a new reward/option is presented). The option’s ‘incentive value’ emerges during this inferential process (inherently relative with respect to reward expectation), and corresponds to what is known as a ‘precision-weighted reward prediction error’. In other words, the incentive value of a choice option corresponds to the change in reward expected in a given context when the option is presented. The prediction error is determined by the difference between the observed and expected reward (derived from integrating expectations within each context). The precision-weighting depends on the prior confidence and optimally (in Bayes sense) normalises the prediction error in relation to uncertainty about both context and rewards (i.e., contexts characterized by a high precision/reliability exert more influence on reward expectancy and the size of these effects decreases with this reward uncertainty). In summary, other options elicit context effects by shaping expectancies before a reward is presented or considered, and people infer, not maximise, the reward available in a given context by combining prior expectations with observations of available rewards (i.e., utility-maximization emerges from this inferential process). This model also postulates that action/choice aims to cancel the prediction error by approach responses/choices elicited by positive errors and avoidance responses elicited by negative errors (this ‘active inference’ process involving prediction error cancelation is assumed to be a product of our biological evolution). In a sense, this model is similar to the adaptive computation framework proposed by Louie, Khaw, and Glimcher [91]—in terms of calculating the value of an choice option by using the statistical distribution of the values of other options in a specific context, but, instead of using a biological mechanism based on division by the pooled activity of other neurons, Rigoli et al. [92,93] ground the model upon simple normative principles of Bayesian statistics (i.e., this model applies to the computational level in Marr’s terminology).

This ‘contextual’ view, as described from multiple levels of explanation, has implications for certain rational choice based disciplines, such as economics where people are assumed merely to have *ordinal* preferences between outcomes—they are assumed not to assign *cardinal* values to outcomes [94]. Thus, the assumption is that people prefer fish to salad, but that they have no grip on *how much* they prefer fish to salad; they do not know, for example, whether they prefer fish to salad more or less than they prefer steak to pasta. Thus, we suggest that the practice in many areas of economics is supported, rather than undercut, by psychological results, but only as long as the set of choice options (products) does not change drastically. If the context (e.g., the set of alternative choice options) *does* change, then this preference ordering may reverse, so that salad is preferred to fish. This contextual view predicts that humans are unable to represent absolute magnitudes, whether perceptual variables, utilities, payoffs, or probabilities, and instead make locally consistent judgments about these quantities based on small samples of available information. Those conclusions lead to a weaker version of economists’ notion of ordinal utility: that ordinal judgments are stable within a context, or sample, containing stimuli from the current environment as well as retrieved from memory. We review evidence that behaviour is consistent *within* contexts and even though it is globally unstable when behaviours across different context are considered (see [95]). We argue that people can only be assumed to have *locally ordinal* (stable and consistent) preferences between outcomes within a particular sampled context. We conclude that economic theory ordinalist assumptions are implausible only when single utility function is used to model choice behaviour across different contexts, in order to create a global preference ordering.

Note, moreover, that, in line with the doubts raised above about the very notion of global rationality, it is by no means clear that a global preference ordering is even conceptually coherent. Consider two contexts, one in which a person chooses *A* over *B*, another in which *B* is chosen over *A*. Is this is an example of irrationality? Or, as the economist and philosopher Sen points out (e.g., [96]), there are endless shifts in context that might justify such choices. Sen notes that a person might choose an apple when the fruit basket is passed around, except when it is the last remaining apple, for reasons of social convention. It is tempting to think that, by incorporating the desire to obey social conventions into our preferences, global consistency can be retained, but there are endless further minor contextual shifts, which may reverse our preferences. For example, suddenly recalling that her companions all dislike apples, or, alternatively, deciding to repay some earlier perceived rudeness, or display authority, might lead the chooser to take the apple after all. Concerns of this type raise significant doubts that there is any stable limit on which the process of joining up local contexts will converge—and hence cast doubt on the very idea of a truly global, i.e., context-independent, preference ordering. 

Moreover, a global preference order appears to require that people must have well-defined and hence consistent beliefs and preferences regarding the credibility of ideas they have never entertained (e.g., degrees of beliefs in as-yet unformulated scientific hypotheses or mathematic conjectures, perhaps stated using theoretical terms that have yet to be invented), and well-defined preferences over options they have never considered (e.g., preferences between as-yet-unread novels or as-yet-un-tasted foods). An agent with a truly global preference ordering, linking together all possible contexts, would have views (however tentative), and hence *preferences* over bets, concerning all possible matters, and these views would need to be consistent. It is unclear whether this viewpoint is even conceptually coherent [19]. 

In summary, our starting assumption is that human thought is about constructing rational arguments in a given context [97], and therefore the algorithm of human decision-making is not inherently irrational as the evidence above might be interpreted to imply. We propose that the human decision algorithm is based on basic cognitive operations, such as inferences based on local comparisons, which provide computational shortcuts to more complex calculations. The psychological explanation also assumes that the value of each alternative is not fixed-and-given, but, instead, it is constructed online from sequential computations of selected pieces of information.

## 5. Local Rationality of Choice II: Empirical Data

What mental process is used each time can often be determined by the input characteristics. Consider a choice among different gambles; if the multiplication of the rewards and probabilities is straightforward, then the trade-off between the two gambles can be reframed on the dimension of “expected value” and the predictions of rational choice theory should coincide with the empirical data. This does not negate our claim that people can use one-mental-process-at-a-time, but it rather demonstrates that the decision process is adaptive and dependent on contextual factors. In summary, contextual shifts can differentiate the valuation of the choice options across different contexts (e.g., [98]), leading to preference reversals that clash with the rational theory, which assumes that rationality holds globally.

Consider, for example, how context shifts are dealt with in prospect theory [99,100], probably the leading descriptive theory of risky choices. Prior to the evaluation phase, the options undergo “editing”, a good example of local rationality. The aim of editing is the reformulation and simplification of the available options in such a way that the subsequent evaluation becomes easier. While in the editing stage, the decision-maker keeps reasoning and reframing the available problem, which ultimately leads to biases due to the limited information used. For example, rounding off the small differences between prospects (i.e., simplification) can create intransitivities. Despite the fact that the reasoning process followed during “editing” is not locally erratic on its own, globally (across different frames) it can lead to violations of rational choice axioms.

The effect of using fragmented information in decision-making has been demonstrated in a study that examined the effect of time-pressure on the magnitude of the compromise effect [101]. The compromise effect refers to the finding that, within a choice set of options that are characterized by two dimensions, alternatives with extreme values are less attractive than alternatives with intermediate values [102]. This effect violates the value maximization principle and indicates the use of a strategy that penalizes extreme options. The aim of Dhar et al. was to examine the effect of time pressure on the magnitude of the compromise effect. If the compromise effect is due to using satisficing heuristics and minimizing cognitive effort, according to the bounded rationality framework, then its magnitude should increase under time pressure. If, on the contrary, the effect occurs because the decision process is stuck in local aspects of the choice problem then its magnitude should diminish under time pressure. The main finding of the study was that increasing the time-pressure reduces the magnitude of the compromise effect. Thus, deliberating for a longer period increases the magnitude of the effect due to an excessive focus on the relational aspects of the alternatives. Despite the fact that the reasoning process is not irrational (e.g., in any given comparison the decision-maker will be attracted more by the best option in the dimension under evaluation), the information that is fed into it is partial and results in biases.

In the face of existing evidence, rationality is best interpreted as a rather local, instead of global, phenomenon, i.e., rationality assumptions typically apply locally, within a context. Note, however, that according to rational choice theory and economics, the appropriate psychological assumption underlying rational explanation is that people will behave consistently within a context (e.g., [103]). The drive to maintain such local consistency resonates with our suggestion that people are ‘locally ordinal’ as shown by Ariely et al.’s [95] suggestion that phenomena such as ‘coherent arbitrariness’ imply that utility maximisation may, in fact, be a valid representation of choices under specific circumstances, without revealing underlying preferences. These results imply that, when people are aware of changes in conditions, such as the change in price, then they will respond in a coherent fashion that mimics the behaviour of individuals with well-defined preferences. Indeed, people’s ability to have stable trade-offs, reason according to the rules of logic and perform probabilistic inference when the context is stable have been demonstrated. For example, experimental economists have consistently shown that choices converge close to rational equilibrium in certain decision situations like first price (English) auctions [104] and second-price auctions after repetition and introspection, in which over-bidding and under-bidding is almost completely eliminated.

However, people may not respond reasonably to new or hidden changes in external variables, or when they lack experience in novel contexts (but if changes are well-publicised, i.e., not hidden, then this could still lead to consistent response in most cases). Our key argument is that rational choice theory is an unrealistic model of human behaviour because the rational models try to hook together (integrate and explain) behaviour across different contexts, in order to create a global preference ordering. This approach does not work in practice because individuals are unstable in their preferences and flip around depending on context. However, the key caveat is that if the distribution of contexts across individuals is reasonably stable, then this would create stable overall behaviour. Analogous phenomena are observed in perception as demonstrated by the well-known *change blindness* phenomenon that occurs when observers do not notice a change in a visual stimulus (e.g., when two images or scenes remain identical apart from a single change). Observers do not notice those changes usually because of lack of attention, eye movements, obstacles in the visual field, or a change of location [67,105,106]. This evidence suggests that the very stability of the visual world (i.e., the mental representations sustaining it) is also dependent on stability of the context or local environment, and, once this stability is removed, our cognitive system cannot maintain even locally consistent representations. In other words, it seems that the human brain is continuously trying to sample and integrate local bits of information, but this process is limited to where attention is focused even within the local scene (see [107,108]). Consistent with this conclusion, experiments show instability in behaviour, and choice preferences in particular, by disconnecting people from their normal environment. For example, the financial value of health-related states, including pain is a critical variable in health economics; however, individuals’ willingness-to-pay (WTP) to avoid painful electric shocks in the laboratory (a rather atypical environment) is subject to substantial local contextual effects based upon the very recent painful experience, the random experimental endowment for each trial, and even beliefs about what other participants consider pain avoidance to be worth [109,110]. There is also evidence for relative valuation of pain in human prefrontal cortex [111]. 

In summary, most of the time, markets may behave stably and locally behaviours can also look stable and consistent. However, as a result of the fundamental instability of mental representations (including cognitions such as perceptions, beliefs, and preferences), such stability is not guaranteed, as we have seen, if the context changes significantly (e.g., if new goods become available, or the market receives an exogenous shock, such as a supply shortage or a sales-tax increase).

## 6. Local Rationality of Choice III: Psychological Theories

Rational models should not be dispensed with—but they need to be supplemented with a theory of contextual shifts. We do not outline the specifics of such a theory here, but instead try to review and summarise the common characteristics of the prominent psychological theories of choice, which are comparative in nature. Hence, we suggest to carry the seed from which such unified theory may grow in the future. The comparative approach does not assume that observable binary choices result from consulting stable (independent) values. This assumption is evident in the fact that revealed preferences work only as long as the set of choice options does not change drastically. If the context (i.e., the set of alternative choice options) does change, then this preference ordering may reverse. Some researchers argue that these effects are due to a human inability to represent absolute magnitudes, whether perceptual variables, utilities, payoffs, or probabilities [69,89]. These results resonate with the idea that preferences are constructed afresh, rather than revealed, in the light of the salient options in each new situation. In the relativistic nature of this account lies implicitly the notion of context as the value of an option changes with contextual shifts.

**Figure 1:**
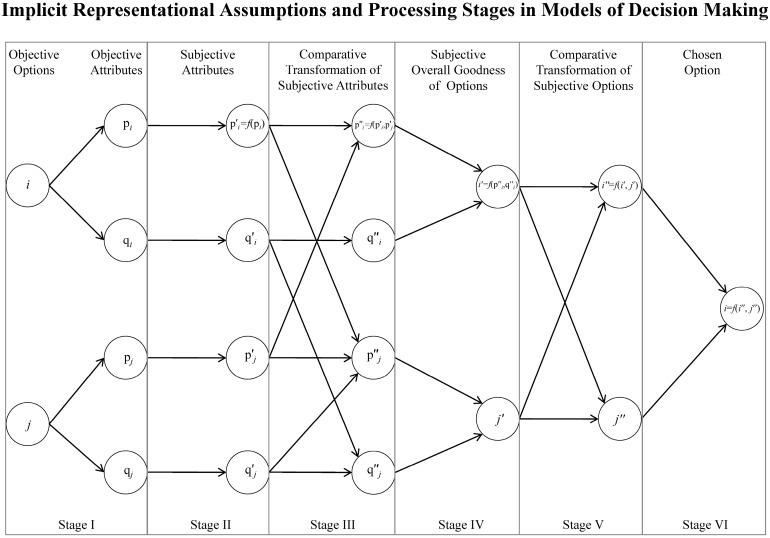
A framework for an abstract decision model, illustrating the processing stages and the representational assumptions of the choice algorithm. The framework incorporates the idea of local processing in stage iii, where the amount of information that is processed for each choice aspect (i.e., dimension) is dependent on the differential weights of each dimension.

Figure 1 presents our generic framework for the abstract description of a decision model, which encompasses the idea of local processing. To simplify our illustration, we assume that the problem at hand is a two-attribute, binary decision. The process comprises of six discrete stages. The first stage involves the objective representation of the attribute values. At the second stage, the objective attributes are transformed through a subjective value function. The third stage computes the relative subjective value of each option in each dimension. The fourth stage integrates the subjective relative values of each dimension, for each option into an overall subjective goodness (subject to differential weighing of the different dimensions). Finally, the fifth and the sixth stages involve the binary comparison of the two options, based on the overall subjective goodness, and the final decision. In stages iii and v, relativistic inference, on some aspects of the decision problem, is performed. Depending on the information that is fed in stage iii, which is subject on the differential weight of each dimension, the inferences operated from the model are potentially *local*. The extension of these abstract representational and processing assumptions, in choice problems with three or more options should be straightforward if one assumes that any multi-alternative process can be broken into elementary binary problems. This reduction makes the decision process even more susceptible to biases since some pairs might be processed more often than others due to selective attention, which is another instance of local processing/inference. Next, we categorize well-known theories of choice into: (a) theories assuming comparative value functions; and (b) theories assuming scale-less comparisons and briefly discuss the way information is processed and context is incorporated (i.e., the representational assumptions and processing stages of Figure 1). This analysis aims to illustrate how most established theories can be described as embodying the local rationality assumptions proposed in this article. The proposed framework also provides taxonomy of psychological models of choice, which indicates that there is a wide variety of ways in which the local nature of rationality can be captured; and by capturing the aspects of the context, these models also provide accounts of how choices change when the context shifts.

### 6.1. Theories Assuming Comparative Local Value Function

After observing that judgments and choices are relative and context-dependent, decision-making researchers have generally assumed that the options are represented on value scales, which are determined by functional transformation of the advantage/disadvantage of each option in relation to all other options. Note that, in value-based models such as prospect theory, the value of each option on each attribute is judged independently of other options, but in relation to a reference point that is unique for each attribute (e.g., money). In contrast, theories assuming comparative value function assume that the reference point for each option is some function of all other options in the set—such relative evaluation means that a value function transforms the ‘relative advantage’ of every choice options on each dimension. 

Therefore, such modern decision theories still assume scales for utility—i.e., attributes have scale-based values that can be compared on an interval scale (i.e., the comparisons are better than ordinal). One of the first models based on that idea was the *componential-context model* [98] of context dependent preference. There the value of an attribute of an option is a function of its magnitude. The value of each option is a weighted sum of its computed advantages/disadvantages (relative to the other options) in each dimension. Additionally, the weighting of each attribute is dependent on the trade-off between attributes in previous choice sets. Thus, to account for context effects (such as reference point effects), objects need not have stable values even though their attributes do. This theory shares all assumptions of Figure 1 except the fifth stage (the comparative transformation of the subjective options) and the effect of the local context is captured in the computation of the advantages/disadvantages (stage iii).

Parducci’s seminal *range-frequency theory* [112] of contextual judgment assumes that a subjective value given to an attribute is a function of its position within the overall range of attributes (measured on an interval scale), and its rank within the current context. Assuming that the range/frequency aspects of the immediate context are encoded in the “comparative transformation” function (stage iii), *range-frequency theory* shares similar representational and processing assumption to the *componential-context model* (stages i, ii, iii, iv, vi).

Similar assumptions (stages i, ii, iii, iv, vi) are shared also by the *stochastic difference model* [113], which assumes a value function that transforms the objective attribute values into subjective ones and a separate function that compares attribute values within a dimension and chooses the highest, only if the difference between two options exceeds a given threshold.

Comparison based value computation, but in a dynamic fashion implemented in a sequential sampling framework, is also assumed by the *multialternative decision field theory* (hereafter MDFT, [114]. At each moment, the decision maker focuses on one dimension of the problem by examining the relevant attribute values of each alternative. Similar to the *componential-context model*, MDFT assumes that the attribute values are objectively defined by their magnitudes. While attention switches stochastically across attributes, a linear value function to evaluate advantages and disadvantages of options on each dimension (relative to other options) provides the basis for subsequent choice, as value differences (or valences) are integrated over time. MDFT incorporates all six stages. In particular, stage (v) (the subjective goodness of options) is factored in by allowing the state of an accumulator (which accumulates relative evidence and tracks the preference state of an option) to be distorted by lateral inhibition from the other competing accumulators. The magnitude of the inhibitory coupling depends on the similarity of two options in the multi-dimensional choice space with the more similar options competing stronger.

The *leaky competing accumulator model* (hereafter LCA, [115]) takes a very similar sequential sampling approach—value is emerging across time by switching attention to different attributes and is represented by activation in neural units (i.e., accumulators), which compete to each other via lateral inhibition. While DFT is a linear model, in the LCA framework, a set of nonlinear assumptions are introduced. The first nonlinearity concerns the dynamics of the model; while DFT does not impose any constraint on the values of the preferences’ states, LCA forces them to be non-negative. This constraint is rather biological since it implies that the preference states correspond to neural firing rates, and thus they cannot be negative. The second nonlinearity is introduced in the value function, with disadvantages having a larger impact than advantages. This loss-aversion property (first introduced in the *componential context model*) is not implemented directly in DFT but instead emerges from the dynamics of the model. Both the *multialternative decision field theory* and *leaky competing accumulator model* assign to context an important role. First, the choice process is inherently local, with attention being allocated to one dimension at each time and second by allowing the options to compete through lateral inhibition, as the decision process evolves. The descriptive adequacy of these models is unique as they are the only models so far able to account for all three preference reversals in multi-attribute choice (i.e., attraction, similarity and compromise effects) simultaneously under single parameter sets. 

Other examples of this approach applied in specific domains have been recently suggested. The *perceived relative argument model* of decision-making under risk [116] was proposed as a descriptive model in which a gamble is preferred over another if each relative advantage in the probability (payoff) exceeds its advantage in payoff (probability) (see also *regret theory*, [117]). The same approach was applied in the *trade-off model* of intertemporal choice in which alternative-based discounting was replaced with attribute-based trade-offs between relative interval differences and payoff differences [118]. Note that the assumption of comparative value models implies the construction of an overall value for each option—but some models assume underlying scales for each attribute but never construct an overall value for each option independently of other options. For example, the difference between value-based and comparative (difference-based) value models is that, while all of these models use value functions to transform attribute values, the latter do not have scales for options [119]. Indeed, most of the *constructed preference approaches* [120] assume scale-based value for various attributes of choice options, which are assembled (constructed) in different manner depending on the context. 

This tradition is followed in neuroeconomics [121], where some researchers argue that evidence suggests a two-stage model in which values are first assigned to goods and actions and then a choice is made from a set [122]. Many other neuroscientists share the view that objects are assigned value and then comparison occurs (i.e., the first stage is pre-comparative) (e.g., [123,124,125]). For example, one such model [126] based on neural data suggests that the brain may not lack absolute value scales, but rather that estimates of value are noisy (as often are sensory percepts). Accordingly, relative comparisons are used to infer distributions of values.

### 6.2. Theories Assuming Scale-Less Local Comparison

The most recent type of comparative theories assume that people make judgments and decisions without consulting a utility scale based on the absolute magnitudes of stimuli (including key “economic” variables such as time and probability). The common feature of this class of models is the lack of value functions operating on (transforming) input variables such as the attribute levels of choice options. Thus, according to these models, ‘direct’ comparison, rather than value, is fundamental to judgment and choice, which implies that absolute differences between attribute levels often do not matter and different processing rules may apply to compare objective values (e.g., numbers) directly (absence of stage ii in Figure 1).

This approach was taken in the *decision-by-sampling* theory [69,127], which assumes that stimuli are judged only on the basis of ordinal binary comparisons with other magnitudes sampled from memory (sampled context) or from the immediate decision context. A counter for each option tracks how many times a binary comparison in a given attribute ended in its favor (i.e., an attribute can be chosen at random, but then that chosen attribute is binarily compared across objects). The option that was the most frequent winner is selected. 

The absence of value-scales may be the reason why people use such a heuristic (see also [112,128]). Other decision rules have also been proposed. For example, in the *priority heuristic* [65], the winning option is chosen by considering only the most important attribute (e.g., minimum gain, probability of minimum gain, or maximum gain) between the available options in the immediate context (i.e., without considering previous contexts). This approach is part of the family of models known as *fast-and-frugal heuristics* [129], which involve lexicographic (in order of importance) application of reasons to eliminate/select choice alternatives. The origins of this approach can be found to earlier heuristic models such as the *elimination by aspects theory* [68]. There the decision process involves the serial application of the following simple non-compensatory (i.e., consideration of one attribute each time) strategic rule; at each time step, one attribute is probabilistically chosen according to its importance and any option that is disadvantageous (below a predefined acceptable threshold) on this attribute is eliminated. If two or more options survive after applying the rule with the highest ranked attribute, then other attributes are considered in order of importance, until one alternative remains. Future research should study how people select the dimension for comparison, which is a key issue for this class of theories. 

The context dependency of such comparative choice strategies makes some of them sensitive to the environmental contingencies encountered by the decision-maker (i.e., the distributions characterizing the sampled context). Due to this property, decision-by-sampling, for example, accounts for typical patterns of economic behaviour like diminishing marginal utility of wealth, losses looming larger than gains, hyperbolic temporal discounting, and overestimation of small probabilities and underestimation of large probabilities. Similarly, the priority heuristic also predicts behavioural phenomena such as Allais paradox, loss aversion, and the certainty effect. These results show how scale-less comparative decision theories, which do not assume any value functions, can explain decision anomalies such as loss aversion.

Another class of models, mentioned here as *reason-based models,* follow a similar logic to scale-less comparative models. In these models, choice emerges from the comparison of the aggregated reasons for and against each option (pros and cons). An early such theory, *reason-based choice* [130] demonstrates how a series of choice anomalies arise when decision-makers resort to the construction or consideration of reasons in order to resolve conflict and to justify their decisions.

Similar in spirit to the *reason-based choice* is *query theory* [131], which offers a memory-based account of endowment effects. According to *query theory*, valuation questions are addressed by serially posing a set of sub-questions whose order differs depending on the response mode. Because earlier queries are weighted stronger, the response mode affects the decision outcome. For example, choosers will first consider reasons why not to enter a transaction and then reasons why to accept it while sellers will follow the opposite order.

*Fuzzy trace theory* [132] also postulates that people are capable of thinking in terms of both exact (i.e., verbatim) and fuzzy (i.e., gist) aspects of choice information with the latter being retrieved from memory (e.g., health-related values, knowledge, experiences) in order to determine the subjective representation of a decision problem. Finally a recent model of *local thinking* [133] demonstrates that a set of decision anomalies might emerge from *local* and *selective* recall of reasons. In summary, reason-based models do not assume any value function, and instead they rely only on counting-up bits of evidence for and against and on explicit thought processes such as ‘arguments’.

## 7. Discussion

The overall argument presented in this article provides, we hope, a fresh perspective on the scope and limits of human rationality in various domains. The aim of this article is to show how the standard focus in rational choice theory on consistency can be derived out of knowledge of basic cognitive mechanisms underlying decision-making, instead of being assumed out of convenience, intuition, and theoretical helplessness. This aspect of cognition is related to how people make inferences that lead to judgments of the magnitudes of choice options, or their attributes, such as values, payoffs, and probabilities, which are essential ingredients of every decision problem. This is a very general and pervasive feature of human cognition that may have important implications for all aspects of decision-making and related fields within psychological and social sciences. Thus, rather than starting from a normative rational theory, such as expectancy-value models of attitudes/action in social psychology [134] and expected utility theory in economics [135], and attempting to make modifications that render it descriptively acceptable, we start from assumptions about elementary cognitive processes, and attempt to construct an account that can address the behavioural phenomenon of choice. That is, our attempt is to bridge from psychology to rational choice theory, and ultimately behavioural sciences, with the ultimate aim of showing that the resulting model predicts decision-making and may still conform to standard assumptions about rationality. In addition, given human inability to make absolute judgment [69], such simple ‘local consistencies’ are possibly the most adaptive cognitive strategies. In addition, indeed, there is some evidence that near-optimal results in various cognitive domains can be obtained by fast and frugal mental heuristics [136,137] and other non-integrative methods like improper linear regression [138] and elimination-by-aspects [68].

### Implications for Understanding Behaviour

Traditional theories of behaviour can be grouped into four categories according to the underlying principles or mechanisms (see [139], for a review). First, most theories can be traced back to the ‘expectancy-value model’ as we discussed before. Second, some theories are purely ‘goal theories’ focusing on the intention-action link, such as goal setting theory [140] and methods such as forming implementation intentions [141], which are plans that specify when, where and how the behaviour will be performed. Third, behavior–attitude theories focus on the feedback loop from action to attitudes, such as Bem’s [84] self-perception theory, which propose that people form attitudes and opinions by observing their behaviour and concluding what mental states must have caused them. Fourth, sociological theories, such as diffusion of innovation theory [142], social consensus model [143], and social ecological model [144], usually do not refer to psychological processes, and focus instead on examining the multiple effects and interrelatedness of social elements in an environment (sometimes at different levels, such as microsystems, mesosystems, and macrosystems), which shape human behaviour.

The first approach is the most predominant view in social, organisational, and health psychology, as well as in public health, medicine, public policy, political science, sociology and anthropology—that behaviour is the result of attitudes and beliefs about the particular actions, outcomes, social approval, control and self-efficacy; and the research agenda in these disciplines is therefore based on more global ‘monolithic’ theories of attitudes and values as stable forces shaping behaviour (e.g., see [48,145]). Humans are assumed to walk around with firm values and propositions (beliefs) in their heads, which they can consistently and reliably access when decision situations prompt them to do so (e.g., when facing choices whether to attend cancer screening, wash their hands, or follow dieting advice). Indeed, this is the ideology behind ‘informed choice’ in public policy, which provides people with information and lets them make up their minds, and expects them to remember and apply all that information and/or experiences in their everyday decision-making (see [146,147]). 

In reality, however, truly informed choice rarely happens; for example, changing intentions (as a results of changes in beliefs and attitudes) accounts for between 3% and 28% of the variance in behaviour change, depending on whether researchers employ experimental [139] or correlational [148] methods, respectively. This is often because a host of situational factors usually determines our causal mental states when we make crucial choices about our health, wealth and well-being [149]. This resonates with recent attempts to characterize a more veridical architecture of human decision-making, which might help shape new policies that *nudge* people to make better decisions in more sophisticated ways than the mere provision of information, in effect by changing the *choice architecture* [150].

The local consistency approach proposed here promises to provide a better account of the predominant view that behaviour is driven by attitudes and beliefs because, if contextual (temporal, spatial, social) ‘localness’ is the key, as we claim, then the information structure of the immediate environment will turn out to be crucial [151]. Our approach implies, therefore, the more realistic view that people predominantly engage in ‘nudged choice’ [150,152,153,154] rather than ‘informed choice’; and that individuals may not have firm attitudes, but instead have to constantly ‘infer’ their attitudes when making everyday decisions (i.e., attitudes are not ‘just there’); and such inferences are local and contextual—people sample what is accessible from memory and the environment at the moment of choice and if they are given a good, rational argument (evidence or information) *when* they make decisions then they might make a good choice, but otherwise some other piece of information will determine their choice. Therefore, even ‘good information’ becomes a nudge when the temporal dimension is taken into account (as in several models discussed before); and if such dynamic cognitive systems can only sample and process limited amount of information, not all relevant information, at the moment of choice, then every choice might become a ‘nudged choice’ (e.g., see [153,155,156]). The third type of behavioural theories—behaviour-attitude theories such as self-perception theory [157], are obviously moving in similar conceptual directions, but perhaps their assumptions are not local enough—not fully recognizing how limited (and how varied, from time to time) the data on which we base our self-perception may be. 

Such conclusions do not imply that people are not, in many ways, quite stable and consistent in their beliefs, desires, attitudes and behaviours, but such stability is manifested probably partly in cases where individuals have a lot of prior evidence about their own behaviours or thoughts. Such stability can also be due to recurrent environments in everyday life, but once the context changes, values and attitudes could easily change too, as demonstrated, for example, by the famous ‘Stanford prison experiment’ and other results supporting situational attribution of behaviour rather than dispositional attribution—i.e., situation causing behaviour, rather than anything inherent in individual personalities (see [158], for a review of related evidence).

Finally, there is a methodological point to be made about what questionnaires and interviews, which are supposed to measure attitudes and beliefs, are actually measuring (see [159]). Are such surveys assuming that respondents retrieve representations of knowledge/beliefs/attitudes or just prompt people to make quick inferences on-the-spot (on the basis of what information they can quickly sample)? The proposed ‘local inference’ framework implies that what these questionnaires often measure is what people think about when they are thinking about these behaviours, but not what they think about when making actual choices—as they may be different information samples in their heads, which could explain the low predictive power of such attitude measures.

## 8. Conclusions

To conclude, rather than viewing psychological data as undermining assumptions made in rational choice theories, which is often the implicit assumption in some psychological theorising, we claim here that, under specific conditions, we can still employ the standard rational choice approach to modelling decision behaviour. However, we also argued that (rational) choice consistency emerges only within a particular context—this is the local consistency hypothesis that people are coherent within each context and rank-correlated across contexts, even though choices appear globally unstable when integrated from different contexts. In this respect, rational choice theories are often imprecise only because they are designed to model behaviour across different contexts, in order to create a global measure of attitudes or preference ordering.
